# Gap junctions in *C. elegans*

**DOI:** 10.3389/fphys.2014.00040

**Published:** 2014-02-11

**Authors:** Karina T. Simonsen, Donald G. Moerman, Christian C. Naus

**Affiliations:** ^1^Department of Cellular and Physiological Sciences, Life Sciences Institute, University of British ColumbiaVancouver, BC, Canada; ^2^Department of Zoology and Michael Smith Laboratories, University of British ColumbiaVancouver, BC, Canada

**Keywords:** *C. elegans*, gap junctions, innexins, intercellular communication

## Abstract

As in other multicellular organisms, the nematode *Caenorhabditis elegans* uses gap junctions to provide direct cell-to-cell contact. The nematode gap junctions are formed by innexins (invertebrate analogs of the connexins); a family of proteins that surprisingly share no primary sequence homology, but do share structural and functional similarity with connexins. The model organism *C. elegans* contains 25 innexin genes and innexins are found in virtually all cell types and tissues. Additionally, many innexins have dynamic expression patterns during development, and several innexins are essential genes in the nematode. *C. elegans* is a popular invertebrate model due to several features including a simple anatomy, a complete cell lineage, sequenced genome and an array of genetic resources. Thus, the worm has potential to offer valuable insights into the various functions of gap junction mediated intercellular communication.

## Gap junctions and innexins

Members of the innexin gene family (innexins: invertebrate analogs of connexins) in *C. elegans* were first described in 1996 (Starich, 1996), and with the complete sequence of the *C. elegans* genome it was revealed that the family consists of 25 genes (*C. elegans* sequencing Consortium, 1998). The *C. elegans* genome, as well as all other invertebrate genomes, contains no connexin homologs, and the innexins share no sequence homology with the connexins. Based on sequence homology with the innexins, a third gene family was identified in vertebrates and named pannexins. Innexins share a similar topology with both connexins and pannexins. This consists of four transmembrane helices, two extracellular loops, and N and C termini located on the cytoplasmic side. Two cysteine residues in each of the predicted extracellular loops are conserved among all innexins. The two extracellular loops of innexins are thought to be longer than in the connexins, i.e., ~50 amino acids for innexins and ~30 amino acids for connexins (Phelan, 2005; Oshima et al., 2013). Further differences were noted when *C. elegans* INX-6 was examined structurally and the molecular dimensions of the junction formed by INX-6 was observed to be greater than those of Cx26; this may be due to greater spacing between adjacent membranes (Oshima et al., 2013).

Gap junctions form when two hemichannels from adjacent cells line up to make a channel; in vertebrates the hemichannels are hexamers of connexin or pannexin proteins and in invertebrates the innexins are also believed to form hexameric channels (Oshima et al., 2013). If the two hemichannels are identical, the gap junction is referred to as homotypic; if the two hemichannels differ, the gap junction is heterotypic. If the hemichannels are composed of only one type of connexin, pannexin or innexin they are called homomeric, and if more than one type of protein is present within the channel, then the gap junction is called heteromeric. In *C. elegans* homotypic gap junctions have been suggested for the majority of innexins studied, namely INX-3, EAT-5, INX-6, INX-16 and INX-19 while UNC-7 and UNC-9 seem to be able to form both homotypic and heterotypic gap junctions. INX-14 and INX-22 are suggested to form heteromeric gap junctions, and these two innexins or perhaps only INX-14 is further suggested to form heterotypic gap junctions with INX-8 and/or INX-9. INX-19 is the only innexin for which a potential function as a hemichannel has been reported (see below for details).

Null and missense mutations are available for all 25 members of the gene family and a range of phenotypes have been reported, including lethality for at least five of the mutant genes, demonstrating the functional importance of gap junctions in the nematode. Unfortunately, detailed functional studies of the *C. elegans* innexins are available for less than half of this protein family.

There are 25 *C. elegans* innexins contributing to gap junctions that are present in virtually all tissue and cell types in the nematode (see Table 1). A comprehensive expression map of all innexins provides potential insights into function (Altun et al., 2009). Promoter regions of all innexins were fused individually to Green Fluorescent Protein (GFP) and visualized throughout the lifespan of the nematodes and a complex and dynamic picture of the expression of innexin genes emerged from this study. Some innexins are expressed mostly during embryogenesis, others mainly in the adult animals and some are constitutively expressed throughout the entire lifespan. Most innexin genes are expressed in multiple tissues, but a few were found to be expressed in only one cell type. This study provides a large amount of detail on innexin expression; however, due to the nature of the experiments using only promoter regions and not full-length protein fusions, not all innexin expression is represented. For example, the authors note that in some cells previously shown to make gap junctions by electron microscopy, no expression of innexins was observed in their study (Altun et al., 2009).

**Table 1:** ***C. elegans* innexins**.

**Gene**	**Sequence**	**Protein size (aa)**	**Expression**	**Phenotype (mutant)**
*inx-1*	C16E9.4	428	Neurons, muscle	–
*inx-2*	F08G12.10	419	Pharynx	–
*inx-3*	F22F4.2	420	Pharynx, neurons	Lethal
*inx-5*	R09F10.4	447	Hypodermis, seam cells, vulval cells	–
*inx-6*	C36H8.2	389	Pharynx	Asynchronous pumping in pharynx
*inx-7*	K02B2.4	556	Pharynx, neurons	Reduced brood size
*inx-8*	ZK792.2	382	Gonadal sheath cells	–
*inx-9*	ZK792.3	382	Gonadal sheath cells	–
*inx-10*	T18H9.5	559	Pharynx, gonadal sheath cells, neurons, vulval cells, muscles	–
*inx-11*	W04D2.3	529	Pharynx, neurons, intestine, vulval cells, muscles	–
*inx-12*	ZK770.3	408	Excretory cell, neurons, glial-like sheath and socket cells, vulval cells	Lethal
*inx-13*	Y8G1A.2	385	Excretory cell, neurons, glial-like sheath and socket cells, vulval cells	Lethal
*inx-14*	F07A5.1	432	Germ cells	Lethal
*inx-15*	R12E2.9	382	Intestine	–
*inx-16*	R12E2.5	372	Intestine	Reduced brood size, constipated
*inx-17*	R12E2.4	362	Intestinal-rectal valve	–
*inx-18*	C18H7.2	436	Neurons, muscles	–
*inx-19 (or nsy-5)*	T16H5.1	454	Neurons	Chemotaxis defective
*inx-20*	T23H4.1	483	Pharynx, intestinal-rectal valve	–
*inx-21*	Y47G6A.1	481	Intestine	–
*inx-22*	Y47G6A.2	462	Germ cells	Lethal
*eat-5*	F13G3.8	423	Pharynx	Asynchronous pumping in pharynx
*che-7 (or inx-4)*	F26D11.10	554	Neurons	Chemotaxis defective
*unc-7*	R07D5.1	522	Neurons, muscles	Uncoordinated movement
*unc-9*	R12H7.1	386	Neurons, muscles	Uncoordinated movement

*Table made using information from Wormbase release 220 (www.wormbase.org) and papers cited within the text*.

All *C. elegans* innexins have orthologs in the sequenced *Caenorhabditis* species *C. briggsae* and *C. remanei* (except *inx-8* and *inx-9* which share a single ortholog in *C. briggsae*), suggesting that all 25 innexins are true genes, not pseudogenes (Altun et al., 2009). Three pairs of the innexins are polycistronic: *inx-12* and *inx-13, inx-16* and *inx-17*, and *inx-21* and *inx-22*. In *C. elegans*, 15% of genes are found in operons, but many operons have been shown to be hybrid operons, meaning that the genes can be co-transcribed as well as being individually transcribed (Blumenthal, 2005). The innexin pairs seem to be expressed in a similar manner; however, they do not seem to give rise to identical phenotypes. For example, RNAi of *inx-16* but not *inx-17* causes a constipated phenotype and *inx-22* but not *inx-21* was identified in screens for repressors of oocyte maturation (Govindan et al., 2006; Peters et al., 2007; Whitten and Miller, 2007). Thus, based on current knowledge for these innexins, it seems likely that they are part of hybrid operons, but the functional importance of this observation is yet to be determined.

Gap junctions permit electrical coupling between cells, as well as passage of small molecules that initiate signal transduction and gene expression. More recently, channel-independent functions have also been proposed (Vinken et al., 2012). In *C. elegans*, electron microscopy has detected gap junctions between neurons with axon-to-axon and axon-to-soma contacts being common and soma-to-soma contacts being less common. Around 10% of the synapses in the *C. elegans* nervous system are comprised by gap junctions and obviously, they are vital for the animal (White et al., 1986). Gap junctions are also present between socket and sheath cells (glial cells), but not between glia and neurons, and between gonadal sheath cells and between oocytes and sheath cells, but not between germ cells and sheath cells in the distal gonad (Hall et al., 1999; Hall and Altun, 2008). Both the pharyngeal muscles and the body wall muscles in *C. elegans* are connected by gap junctions. Electrical coupling between body wall muscles is what allows for the coordination in the sinusoidal movement of nematodes. The intestinal cells also form gap junctions with one another and the hypodermis makes several gap junctions to neighboring tissues, including seam cells and the excretory canal (Hall and Altun, 2008; Peters et al., 2007; Altun et al., 2009). Clearly, gap junctions are important in *C. elegans*; however, the specific innexins comprising the gap junctions are far from fully understood. Below we summarize what is known about specific innexin genes.

## *C. elegans* innexins

### INX-3

INX-3 was the first *C. elegans* innexin shown to form an intercellular channel. Expression of INX-3 in *Xenopus* oocytes enabled electrical coupling between paired oocytes. Notably, the gating properties were indistinguishable from those of connexins (Landesman et al., 1999). Light microscopy shows INX-3 at plaque-like structures at cell-cell boundaries, as expected for gap junctions, and electron microscopy confirms that INX-3 is indeed part of gap junctions in *C. elegans*. INX-3 is expressed throughout embryonic development and the protein is essential for survival in *C. elegans*. Most *inx-3* mutants die during embryogenesis and the few larvae that hatch never make it past the L1 stage (see Box 1). The hatched larvae have short or sometimes a detached pharynx and the pumping in pharynx is unsynchronized, like mutants lacking *eat-5* and *inx-6* (see below). In the adult worm, the INX-3 protein can only be detected in the posterior pharynx. Overexpression of *inx-3* causes terminal defects during embryogenesis similar to the ones observed in the *inx-3* mutant, so obviously this innexin is crucial for this developmental process, but the exact function remains unknown (Starich et al., 2003).

Box 1 ***C. elegans*—an excellent model organism.**

*C. elegans* is a small (~1 mm), non-parasitic, transparent nematode with a fairly simple anatomy and the adult animal has only 959 somatic cells. It is easily cultured in the laboratory on agar plates seeded with *E. coil* bacteria and it grows from embryo to fertile adult in approximately 3 days at 20° C. The worm goes through four larval stages, L1–L4, and then reaches adulthood. Limitations in food or other stress factors at the L1–L2 stage causes the larvae to enter dauer stage, a stress-resistant, and long-lived diapause stage. When food becomes available, the dauer will resume development. The total lifespan of *C. elegans* is around 3 weeks.

The animal is hermaphroditic and self-fertilizing, as it produces both oocytes and sperm. Hermaphrodites have two X chromosomes, while males have a single X chromosome. Male and hermaphrodite crosses can be done for genetic studies. Adult hermaphrodites, when self-fertilizing, will produce around 300 progeny. Mutant animals can be obtained by chemical mutagenesis and strains can be stored frozen for extended periods of time (decades). Many mutants can be maintained as self-fertilizing hermaphrodites on plates. Transgenic animals, for example animals expressing a GFP fusion protein, can be generated by microinjection or bombardment of exogenous DNA. RNA-mediated interference (RNAi) is readily obtained in *C. elegans* by feeding worms *E. coil* expressing dsRNA corresponding to the gene of interest. 40% of *C. elegans* genes have human orthologs (greater than 7000 genes).

*C. elegans* feeds by pumping bacteria into the pharynx, a tube-like muscular pump with its own nervous system. Bacteria trapped in the pharynx are broken up by muscle contractions and passed into the intestine, the digestive organ extending almost the entire length of the animal. The nervous system of the worm is comprised of 302 neurons and the complete wiring is known. Most neurons are located in the head of the worm, and cells of the nervous system are organized into ganglia in the head and tail. Body wall muscles run along the whole length of the animal in four quadrants and smaller muscles are located in the pharynx and around the intestine and the vulva. The reproductive system in the hermaphrodite consists of two gonadal arms joined by a shared uterus. Germ cells in the distal part of the gonad are undifferentiated and mature as they move proximally. The mature oocytes are ovulated into the sperm-containing spermatheca, where they are fertilized before moving into the uterus. Figure adapted from www.wormatlas.org.

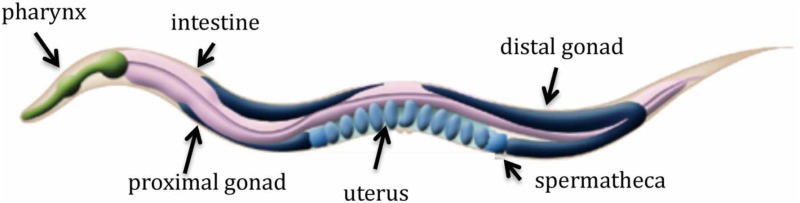

### Eat-5 and INX-6

*eat-5* mutants were named based on their abnormal eating; they display unsynchronized muscle contractions in the pharynx, the organ responsible for capturing bacterial food, grinding it up and transporting it to the intestine (see Box 1) (Starich, 1996). The pharyngeal muscles are electrically coupled by gap junctions, much like cardiac muscle cells, and in *eat-5* mutants, injected carboxyfluorescein fails to spread into adjacent muscle cells of the pharynx (Starich, 1996), suggesting that the gap junctions are formed by EAT-5. An attempt to induce electrical coupling in *Xenopus* oocytes with *eat-5* failed, however, and no coupling could be detected even when *eat-5* was co-expressed with *inx-3*, an innexin with partially overlapping expression with *eat-5* (Landesman et al., 1999). This does not rule out that EAT-5 forms genuine gap junctions, since the observed lack of coupling could be due to lack of the right innexin partner being present, or the channel formed may not be active due to lack of post-translational modification. Inability to couple *Xenopus* oocytes has also been observed for a few connexins e.g., connexin33 and connexin31.1 (Landesman et al., 1999).

*inx*-6 is another innexin expressed in the pharyngeal muscles in a similar pattern to *eat-5*, and, as for *eat-5*, synchronized muscle contractions in the pharynx is lost in this mutant (Li et al., 2003). The *inx-6* mutant animals also display similar defects in dye spreading as that observed for the *eat-5* mutant, and interestingly, EAT-5 could partially substitute for INX-6 function, hinting at very similar, though not identical, roles for these two innexins. *inx-6* is a cold-sensitive mutant and at the restrictive temperature it arrests development after hatching. This strong phenotype is due to a point mutation in a non-conserved area in the C terminus of the protein, suggesting this intracellular region is crucial for correct protein function.

Recently, recombinant INX-6 channels were purified and electron microscopy revealed loosely packed hexagonal gap junction plaques with a larger channel diameter than that observed for connexins (~140 Å for INX-6 as compared with 92 Å for connexin26). Microinjection of low molecular weight fluorescent dyes indicated that the INX-6 channel is more permeable than connexin43, as the INX-6 channels allowed passage of 3 kDa and even some 10 kDa tracers. Curiously, INX-6 formed gap junctions when expressed in the insect cell line Sf9 but did not form gap junctions in mammalian HeLa cells, which could reflect the need of a modifier or modification for INX-6 to form actual channels (Oshima et al., 2013).

### INX-16

The innexin gene *inx-16* is expressed in the intestine and the protein localizes to cell-cell contacts between intestinal cells, consistent with this innexin being a gap junction protein. Observations in a mosaic worm (in which some cells are wild type and some cells are mutant) suggest that INX-16 forms homotypic channels, since the protein is absent from cell-cell contacts between *inx-16* expressing and non-expressing intestinal cells (Peters et al., 2007). The mutant is constipated and, presumably as a result of this, small, slow growing and gives rise to fewer progeny than wild type *C. elegans*. INX-16 allows Ca^2+^ waves to travel between intestinal cells and since this calcium flux is required for intestinal muscle contractions, the mutant's defecation cycle is disrupted, leaving the animal constipated. The absence of Ca^2+^ flux in the intestine was also observed in a different study where the mutant blocked the spread of an endogenous fluorescent marker in the intestine, a phenomenon shown to be preceded by a Ca^2+^ wave (Coburn et al., 2013). Dye transfer of Lucifer yellow between the intestinal cells was not inhibited in the *inx-16* mutant (Peters et al., 2007), but other innexins are also likely to be expressed in the intestine, including *inx-2*, *inx-11* and *inx-15* (Altun et al., 2009). Despite the potential for several gap junctions to function in the intestine, INX-16 is clearly necessary for allowing Ca^2+^ waves to propagate throughout the intestine.

### INX-22 and INX-14

The adult hermaphrodite gonad consists of two U-shaped arms that share a common uterus; the germ cells in the distal gonad are undifferentiated and as they move to the proximal gonad (closest to the uterus) they mature and the oocytes will move into the sperm-containing spermatheca, where they are fertilized (see Box 1). Subsequently they move into the uterus and ultimately, the eggs are laid through the vulva. Gonadal sheath cells (myoepithelial cells) cover the proximal gonad arm and most of the distal gonad arm; they form gap junctions with each other and with the proximal germline, as has been shown by electron microscopy (Hall and Altun, 2008). INX-14 and INX-22 are expressed in the germ cells and co-localize in plaque-like structures at the interface between sheath cells and oocytes in the proximal and distal gonad, consistent with these two innexins forming gap junctions (Govindan et al., 2009). In an *inx-22* null mutant, immunostaining showed that INX-14 was absent from the proximal gonad, but interestingly, INX-14 localized almost normally in the distal gonad (Edmonds et al., 2011). One interpretation of these results is that in the proximal gonad, INX-14 and INX-22 form heteromeric gap junctions. Null mutation in *inx-14* causes sterility due to defects in oogenesis (Govindan et al., 2009), but null mutation in *inx-22* does not show this phenotype; these mutants are fertile, although they have reduced brood size. This is the expected result if these proteins act together in the proximal gonad but redundantly in the distal gonad.

These two innexins were both identified as repressors of oocyte maturation in the absence of sperm [in a *fog-2(q71)* background, which generates XX females and XO males] (Govindan et al., 2006; Whitten and Miller, 2007). In *C. elegans* the process of meiotic maturation is tightly coupled to sperm availability with sperm secreting the signaling molecule major sperm protein (MSP), which acts as a paracrine hormone and induces oocyte maturation and sheath contraction. The extracellular MSP gradient in the gonad of hermaphrodites is not believed to be high enough to reach the loop region of the gonad where stimulation of oocyte maturation takes place, and the sheath cells are hypothesized to communicate the presence of the MSP signal throughout the whole germline since the sheath cells are coupled to one another and to the germline via gap junctions. The two screens identifying *inx-14* and *inx-22* as negative regulators of oocyte maturation were both RNAi screens and would not have picked up any innexins with redundant functions (Whitten and Miller, 2007).

Further functional studies highlight differences between INX-14 and INX-22; INX-14 is required to inhibit oocyte maturation only in the absence of sperm, INX-22 is required in the presence and absence of sperm (Whitten and Miller, 2007). INX-14, but not INX-22, was also shown to be important in the process of guiding male sperm, deposited during mating, from the vulva to the spermatheca, where fertilization takes place. Absence of INX-14 causes defects in velocity and directionality of sperm migration (Edmonds et al., 2011). Also in this process, INX-14 was shown to function within the germ cells, so in order to identify potential innexins partner(s) in the sheath cells the four innexins shown to be expressed in the gonadal sheath cells (*inx-5*, *inx-8*, *inx-9*, and *inx-10*) (Altun et al., 2009) were tested, and RNAi of *inx-8* and *inx-9* showed similar sperm guidance defects. As these genes are 85% identical, RNAi of either gene is predicted to target both genes, so the results for these two innexins cannot be separated. Oocytes secrete prostaglandins to guide sperm to the spermatheca and signaling via innexins in gap junctions is suggested to control prostaglandin transport and/or activity (Edmonds et al., 2011).

Taken together, signals from the gonadal sheath cells to the germ cells seem to be communicated via gap junctions made of the innexins INX-14 and INX-22 and most likely INX-8 and INX-9, with the possibility of even more innexins being involved. Interestingly, the gene *inx-14* was also identified in a forward genetic screen for mutants resistant to the pathogenic bacteria *S. aureus*. Subsequently, *inx-8* and *inx-9* were also tested and they too showed resistance (Miyata et al., 2008).

### UNC-7 and UNC-9

Mutants of the genes *unc-7*, the first innexin identified in *C. elegans*, and *unc-9* display identical uncoordinated movement phenotypes often referred to as kinking: the animals move in very severe bending motions instead of the normal sinusoidal body bends as generated in wild type nematodes. UNC-7 and UNC-9 are both expressed throughout the nervous system as well as in body muscles. There is extensive co-localization of these two proteins in the nervous system, but in body wall muscles UNC-9 is reported to be expressed at a higher level than UNC-7. These expression data used an *unc-9::gfp* construct which only partially rescued the uncoordinated phenotype, maybe due to GFP interfering with UNC-9 function (Starich et al., 2009). Both innexins are able to electrically couple paired *Xenopus* oocytes and they both form homotypic and heterotypic gap junctions, but no evidence for heteromeric gap junctions was found (Starich et al., 2009). Since removal of functional UNC-7 or UNC-9 in muscles does not lead to an uncoordinated phenotype, it is suggested that these two innexins are required in the nervous system for coordinated locomotion.

Despite the similar phenotypes of mutants in *unc-7* and *unc-9* and similar expression patterns of the two genes, functional studies show large differences between the two innexins. Body wall muscles in *C. elegans* are electrically coupled via gap junctions and this coupling is significantly reduced in the *unc-9* mutants, but not in the *unc-7* mutant (Liu, 2006). Remarkably, five other innexins have been shown to also contribute to the electrical coupling in the body wall muscles: INX-1, INX-10, INX-11, INX-16, and INX-18 (Liu et al., 2013). The stomatin-like protein UNC-1 is also involved in this electrical coupling of the body wall muscles in *C. elegans*, and UNC-1 co-localizes with UNC-9 at intercellular junctions in body wall muscles. Bimolecular fluorescence complementation assays confirmed that these two proteins are in very close proximity (Chen et al., 2007). Localization of UNC-9 is not dependent on UNC-1, nor is UNC-1 dependent on UNC-9 for guidance or localization. This has led these authors to speculate that the primary function of UNC-1 may be to modulate the gating of gap junctions. This is the only study in *C. elegans* to possibly identify an interacting partner for one of the innexins. UNC-9 and UNC-1 are both required for synchronized action potentials and Ca^2+^ transients between neighboring body wall muscle cells, demonstrating that UNC-9 gap junctions together with UNC-1 are involved in synchronizing muscle activities (Liu et al., 2011).

### INX-19 (NSY-5)

The gap junction protein INX-19 (also known as NSY-5, for neuronal symmetry) is expressed in a subset of neurons in the head and tail and was identified in a genetic screen for mutants lacking asymmetrical AWC olfactory neurons. The pair of AWC neurons are morphologically symmetric, but they differ in their ability to sense different chemicals due to different genes being expressed in either the left or right neuron (Chuang et al., 2007). INX-19 can electrically couple *Xenopus* oocytes and measurements in the *Xenopus* system also showed a hemichannel action for this innexin, thus INX-19 may be able to form gap junctions and perhaps even functional hemichannels (Chuang et al., 2007). Expression was observed during embryogenesis, with the highest levels of INX-19 in late embryogenesis and at the L1 larval stage; in the adult worm only weak expression was observed in several neurons excluding the AWC olfactory neuron pair. Serial-section electron microscopy on embryos and L1 larvae revealed extensive gap junction coupling between cell bodies of the AWC neurons and other neurons, and in mutants these gap junctions are missing. The gap junctions are not present in adult worms (Chuang et al., 2007). Dye transfer assays using a caged photoactivatable fluorescent dye in isolated embryonic neurons containing either a wild-type *inx-19* gene or an *inx-19* loss-of function mutation demonstrated that INX-19 is crucial in these neurons for dye transfer (Schumacher et al., 2012). Collectively, this is strong evidence that INX-19 forms functional gap junctions allowing for the passage of small molecules between neurons in the developing nematode. The sub-cellular location of this innexin was further studied by expressing *inx-19* fused to GFP in COS cells (fibroblast-like cell line derived from monkeys), showing that INX-19 localized to plaques at cell-cell contacts when both cells were expressing this innexin, suggesting the formation of a homotypic gap junction (Chuang et al., 2007).

In wild type animals, one AWC^ON^ and one AWC^OFF^ neuron are always generated, where the ON and OFF denotes the expression of the reporter *str-2::gfp*. Removal of *inx-19* caused two AWC^OFF^ neurons, whereas overexpression of *inx-19* generated two AWC^ON^ neurons, clearly showing the importance of this innexin in establishing the asymmetry of the AWC neurons. By expressing vertebrate Ca^2+^-buffer proteins within the INX-19 network, the AWC asymmetry was disrupted, thereby indicating that Ca^2+^ signaling is involved in establishing the left-right asymmetry in *C. elegans* AWC olfactory neurons via an INX-19 transient gap junctional network (Schumacher et al., 2012).

## Potential of *C. elegans* as a model for the study of gap junctions

*C. elegans* has 25 innexins and, so far, in-depth functional analysis has been done for less than half of the genes including *inx-3*, *inx-6*, *eat-5*, *inx-16*, *inx-14*, *inx-22*, *inx-19*, *unc-7*, and *unc-9*. All these innexins seem to act as genuine gap junctions, as evidenced by a variety of techniques; electron microscopy, *Xenopus* oocyte coupling, expression of innexins in COS cells and Sf9 cells, fluorescent dye transfer assays, studies of GFP fusion-proteins, voltage clamp and RNAi. One of the most interesting results to come from these studies was the identification of a modifier of gap junctions formed by UNC-9, namely the stomatin-like protein UNC-1 (Chen et al., 2007). There has been substantial work done in identifying connexin interacting proteins/modifiers (Laird, 2010), and this area may hold many more exciting findings for *C. elegans* innexins. Another important finding is that channels formed by *C. elegans* INX-6 show a more permeable gap junction with a larger channel diameter than has been reported for connexins (Oshima et al., 2013). The implications of this observation are not clear at this time. Several interesting observations pertaining to the role of innexins in the biology of *C. elegans* have emerged from these studies including roles in movement, fertilization, oocyte maturation, cell identity and innate immunity. In other words, innexins are involved in almost all aspects of the development of this organism.

When studying gap junctions, dye transfer assays and specific inhibitors are often used, and in *C. elegans* these approaches have been used in various cell types. When carboxyfluorescein was injected into muscle cells of dissected pharynxes, it spread to the remainder of pharyngeal muscles in wild type worms, but not in *eat-5* mutant animals (Starich, 1996). Another fluorescent dye, Lucifer yellow, was injected into the intestinal cells of *C. elegans* and here the dye transferred throughout the intestine (Peters et al., 2007). Carboxyfluorescein was also injected into body wall muscle cells, but here the dye did not diffuse into neighboring cells (Liu, 2006). Isolated embryonic neurons were cultured and loaded with an photoactivatable (or caged) fluorescent dye, which after uncaging by UV light travelled to neighboring cells through gap junctions made of INX-19 (Schumacher et al., 2012). The gap junction blocker carbenoxolone and the pannexin blocker probenecid were tested on UNC-9 in body wall muscles, but none of these chemical inhibitors showed any effect (Liu et al., 2011).

Although the vertebrate connexins and invertebrate innexins are completely unrelated by sequence, they are both the molecular building blocks of gap junctions and thus enable intercellular communication. Much is still unknown for innexins—Are they post-translationally modified? Can they form functional hemichannels? Do any of them have channel-independent functions? Perhaps the biggest question still to be answered is, what small molecules are transported through the innexin gap junctions? *C. elegans* is a small animal model with advantages for studying cell-cell communication, and this nematode has the potential to contribute a great deal to our understanding of how gap junctions function.

### Conflict of interest statement

The authors declare that the research was conducted in the absence of any commercial or financial relationships that could be construed as a potential conflict of interest.
